# A Weak Selection Stochastic Gradient Matching Pursuit Algorithm

**DOI:** 10.3390/s19102343

**Published:** 2019-05-21

**Authors:** Liquan Zhao, Yunfeng Hu, Yanfei Jia

**Affiliations:** 1 Key Laboratory of Modern Power System Simulation and Control & Renewable Energy Technology, Ministry of Education (Northeast Electric Power University), Jilin 132012, China; huyunfeng22@163.com; 2 College of Electrical and Information Engineering, Beihua University, Jilin 132013, China; jia_yanfei@163.com

**Keywords:** compressed sensing, low rank matrix, stochastic gradient, weak selection method, reliability verification strategy, reconstruction performance

## Abstract

In the existing stochastic gradient matching pursuit algorithm, the preliminary atomic set includes atoms that do not fully match the original signal. This weakens the reconstruction capability and increases the computational complexity. To solve these two problems, a new method is proposed. Firstly, a weak selection threshold method is proposed to select the atoms that best match the original signal. If the absolute gradient coefficients were greater than the product of the maximum absolute gradient coefficient and the threshold that was set according to the experiments, then we selected the atoms that corresponded to the absolute gradient coefficients as the preliminary atoms. Secondly, if the scale of the current candidate atomic set was equal to the previous support atomic set, then the loop was exited; otherwise, the loop was continued. Finally, before the transition estimation of the original signal was calculated, we determined whether the number of columns of the candidate atomic set was smaller than the number of rows of the measurement matrix. If this condition was satisfied, then the current candidate atomic set could be regarded as the support atomic set and the loop was continued; otherwise, the loop was exited. The simulation results showed that the proposed method has better reconstruction performance than the stochastic gradient algorithms when the original signals were a one-dimensional sparse signal, a two-dimensional image signal, and a low-rank matrix signal.

## 1. Introduction

Compressed sensing (CS) [1,2,3,4] has been receiving considerable attention. The main premise of CS theory is that the reconstruction of a high-dimensional sparse (or compressive) original signal from a low-dimensional linear measurement vector under the measurement matrix should satisfy the restricted isometry property (RIP) [5]. At present, CS is divided into the following three core aspects: Sparse representation of the signal, nonrelated linear measurements, and signal reconstruction. The sparse representation of the signal is used as the design basis for the over-complete dictionary [6,7] with the capability of sparse representation, such as discrete cosine transform (DCT), wavelet transform (WT), and Fourier transform (FT). These functions are used as the sparse representation of the signal, where they obtain a fine effect. Unrelated linear measurement is used to design the measurement matrix [8] that satisfies the RIP condition. The commonly used measurement matrices include the Gaussian random matrix, the Bernoulli random matrix, and the partial Hadamard matrix. In this study, we focused mainly on the signal reconstruction.

Signal reconstruction methods can be divided into two categories: Those based on the minimized $l_{1}$-norm problem, and the greedy pursuit algorithm based on the minimized $l_{0}$-norm problem. Those in the first category include methods such as the basis pursuit (BP) [9] algorithm and its optimization algorithm, the gradient projection for sparse reconstruction algorithm (GPSR) [10], the iterative threshold (IT) [11], the interior point method [12], and the Bergman iteration (BT) [13] method. These algorithms are generally used to solve the convex optimization problems. The convex optimization algorithms have a better reconstruction performance and theoretical performance guarantees; however, they are sensitive to noise and usually suffer from heavy computational complexity when processing large signal reconstruction problems. The second category includes methods such as the matching pursuit (MP) [14], orthogonal matching pursuit (OMP) [15], regularized OMP (ROMP) [16], and stage-wise OMP (StOMP) [17]. These algorithms offer much faster running times than the convex optimization methods, but they lack comparable strong reconstruction guarantees. Greedy pursuit algorithms, such as subspace pursuit (SP) [18], compressive sampling matching pursuit (CoSaMP) [19,20], and iterative hard threshold (IHT) [21] algorithms, have faster running times and essentially the same reconstruction guarantees, but these algorithms are only suitable for one-dimensional (1D) signals in compressed sensing.

Several algorithms that are deemed suitable for a 1D signal and multidimensionality signals have been proposed. Ding et al. [22] and Rantzer et al. [23] proposed the forward selection method for sparse signal and low rank matrix reconstruction problems. The algorithm iteratively selects each nonzero element or each rank-one matrix. Wassell et al. [24] proposed a more general sparse basis based on previous studies [22,23]. Liu et al. [25] proposed the forward-backward method, where the atoms can be completely added or removed from the set. Bresler et al. [26] extended this algorithm beyond the quadratic loss studied in Liu et al. [25]. Soltani et al. [27] proposed an improved CoSaMP algorithm for a more general form objective function. Bahmani et al. [28] used the gradient matching pursuit (GradMP) algorithm to solve the reconstruction problem of large-scale class signals with sparsity constraints based on the CoSaMP algorithm. However, for large-scale class signal reconstruction problems, the GradMP algorithm needs to compute the full gradient of the objective function, which greatly increases the computation cost of the algorithm. Therefore, Needell et al. [29] proposed a stochastic version of the GradMP algorithm that was called the StoGradMP algorithm. Compared with the GradMP algorithm, the StoGradMP algorithm randomly selects an index and computes its associated gradient at each iteration. This operation is extremely effective for large-scale signal recovery problem.

Although the StoGradMP algorithm effectively reduces the computational cost of the algorithm, its reconstruction capability still needs improvement. In the StoGradMP algorithm, the atomic selection method of the fixed number (namely, selecting 2*K* atoms to complete the expansion of the preliminary atomic set at each round of iterations) leads to a preliminary atomic set of the existing atoms that cannot be fully matched with the original signal. When these atoms are added to the candidate atomic set, the accuracy of the least square solution and the inaccuracy of the support atomic set are affected, which then weakens the reconstruction capability of the signal and increases the computational complexity of the StoGradMP algorithm. Therefore, in this study, we created a weak selection threshold method to select the atoms that best match the original signal, thereby completing the expansion of the preliminary atomic set with a more flexible atom selection. This method improves the reconstruction performance of the algorithm. The combination of the two reliability guarantee methods ensures the correctness and effectiveness of the proposed algorithm, identifies the support atomic set, and calculates the transition estimation of the original signal. Finally, we established different original signal environments to verify the reconstruction performance of the proposed method.

The layout of this paper is as follows. Section 2 introduces the CS theory for signal reconstruction and low-rank matrix reconstruction. The StoGradMP algorithm is described in Section 3. The proposed method, with the weak selection threshold method and the reliability verification strategy of the stochastic gradient algorithm, are outlined in Section 4. The simulation results and the discussion are provided in Section 5, and the conclusion is drawn in Section 6.

## 2. Compressed Sensing Theory

CS theory supposes that signal *x* is an *n*-length signal. It is said to be a *K*-sparse signal (or compressive) if *x* can be well approximated using K coefficients under some nonrelated linear measurements. According to the CS theory, such a signal can be acquired by the following linear random projection:

(1) u=Φx+ε

where $\Phi \in R^{m\times n}$, $u\in R^{m\times 1}$(m≪n), and $\varepsilon \in R^{m\times 1}$ are the measurement matrix [30], the observation vector, and the noise signal, respectively; u contains nearly all the information of the sparse signal x. According to Equation (1), the dimensionality of u is much lower than the dimensionality of x. This problem is an underdetermined problem, which shows that Equation (1) has an infinite number of solutions. It is difficult to reconstruct the sparse signal vector x from u. However, according to the literature [5,31], a sufficient condition for exact signal reconstruction is that the sensing matrix Φ should satisfy the RIP condition. The RIP condition is described in Definition 1.

**Definition** **1.** *For each integer* K=1,2,…, *define the restricted isometry constant* $\delta_{K}$ *of the sensing matrix* Φ *as the smallest number, such that holds for all* K *-sparse signal vectors* $x\in R^{n\times 1}$ *with* ${\left\|x\right\|}_{0}=K$ *.*

(2) $$\left(1-\delta_{K}\right){\left\|x\right\|}_{2}^{2}\leq {\left\|\Phi x\right\|}_{2}^{2}\leq \left(1+\delta_{K}\right){\left\|x\right\|}_{2}^{2}$$

Assuming that the original signal x is sparse in compressed sensing, then x can be reconstructed by solving the following optimization problem:

(3) $$\underset{x\in R^{n\times 1}}{\min}\frac{1}{2m}{\left\|u-\Phi x\right\|}_{2}^{2}\;\mathrm{Subject}\;\mathrm{to}\;{\left\|x\right\|}_{0}\leq K$$

where *m* is the number of the measurement value and ${\left\|.\right\|}_{2}^{2}$ denotes the square of the 2-norm of the noise signal estimate vector. Here, K controls the sparsity of the solutions to Equation (3).

The low-rank matrix reconstruction problem can be similarly formulated. We obtain the observation vector $u_{i}$, which can be described as

(4) $$u_{i}=\Phi_{i}X+\varepsilon_{i}$$

where i=1,2,…,m, the size of measurement matrix $\Phi_{i}$ is an $m\times n_{1}$; the unknown signal matrix $X\in R^{n_{1}\times n_{2}}$ is assumed to be a low rank matrix; and $\varepsilon_{i}$ is the measurement noise. According to Equation (4), the matrix *X* can be reconstructed by the solving the following optimization:

(5) $$\underset{X\in R^{n_{1}\times n_{2}}}{\min}\frac{1}{2m}{\left\|u-\Phi X\right\|}_{2}^{2}\;\mathrm{Subject}\;\mathrm{to}\;\mathrm{rank}(X)\leq R$$

where m is the number of the measurements; u, Φ, and X are the observation signal, measurement matrix, and low-rank matrix signal, respectively; and R controls the rank level of the solution to Equation (5).

To analyze Equations (3) and (5), we first define a more general notion of sparsity. Given the sparse basis $\Psi =\{\psi_{1},\psi_{2},\ldots ,\psi_{n}\}$, which consists of the vectors $\psi_{i}$

(6) $$x=\sum_{i=1}^{n}\alpha_{i}\psi_{i}=\Psi \alpha$$

where $\alpha_{i}=\langle x,\psi_{i}\rangle =\psi_{i}^{T}x$ is the projection coefficient of the original sparse signal x and K≪n. x is sparse with respect to the sparse basis Ψ if the number of nonzero entries are much lower than the length of signal x; that is, K≪n. The sparse basis Ψ can be explained respectively:

(1) For sparse signal reconstruction, the sparse basis Ψ could be a finite set, such as $\Psi ={\left\{\psi_{i}\right\}}_{i=1}^{n}$, where $\psi_{i}$ is the basic vector in the Euclidean space.
(2) For low-rank matrix reconstruction, the sparse basis Ψ could be an infinite set, such as $\Psi ={\left\{\varphi_{i}\nu_{i}\right\}}_{i=1}^{\propto }$, where $\varphi_{i}\nu_{i}$ are the unit-norm rank-one matrices.

This notion is sufficiently general to address several important sparse models, such as the group sparsity and low ranks [28,32]. Therefore, we can describe Equations (3) and (5) using Equations (7) and (8), respectively:

(7) $$\underset{x}{min}\underset{F\left(x\right)}{\underbrace{\frac{1}{M}\sum_{i=1}^{M}f_{i}\left(x\right)}}\;\mathrm{Subject}\;\mathrm{to}\;{\left\|x\right\|}_{0,\Psi }\leq K$$

(8) $$\underset{X}{min}\underset{F\left(X\right)}{\underbrace{\frac{1}{M}\sum_{i=1}^{M}f_{i}\left(X\right)}}\mathrm{Subject}\;\mathrm{to}\;\mathrm{rank}{\left(X\right)}_{\Psi }\leq R$$

where $f_{i}\left(x\right)$ is the smooth function, which can be a non-convex function; ${\left\|x\right\|}_{0,\Psi }$ controls the sparsity level of signal; $f_{i}\left(X\right)$ is also the smooth function with respect to the low rank matrix, which is the non-convex function; and $\mathrm{rank}{\left(X\right)}_{\Psi }$ determines the rank level of the low rank matrix X. In particular, ${\left\|x\right\|}_{0,\Psi }$ is the smallest number of atoms in Ψ, such that the original signal x can be described by

(9) $${\left\|x\right\|}_{0,\Psi }=\underset{x}{\min}\left\{K:x=\sum_{i\in \left|E\right|}\alpha_{i}\psi_{i},\left|E\right|=K\right\}$$

where |E| denotes the number of nonzero entries in the original signal x.

According to Equations (7) and (8), the reconstruction problem of the sparse signal and the low rank matrix need to be separately explained.

For the sparse signal reconstruction, the sparse basis Ψ consists of n basic vectors, each of size n in Euclidean space. This problem can be regarded as a special case of Equation (7), where $f_{i}\left(x\right)={\left(u_{i}-\langle \varphi_{i},x\rangle \right)}^{2}$ and M=m. In this case, we need to decompose the observation signal u into a non-overlapping vector $u_{b_{i}}$ of size b. The matrix $\Phi_{b_{i}}$ is the sub-matrix of size $b_{i}\times n$, which consists of partial row vectors in the measurement matrix Φ.

According to Equations (3) and (7), the smooth function is $F\left(x\right)=\frac{1}{2m}{\left\|u-\Phi x\right\|}_{2}^{2}$. Therefore, the smooth function F(x) can be written as

(10) $$F\left(x\right)=\frac{1}{M}\sum_{i=1}^{M}\frac{1}{2b}{\left\|u_{b_{i}}-\Phi_{b_{i}}x\right\|}_{2}^{2}=\frac{1}{M}\sum_{i=1}^{M}f_{i}\left(x\right)$$

where M=m/b, representing the number of the sub-matrix M, is an integer. Consequently, each sub-function $f_{i}\left(x\right)$ can be treated as $f_{i}\left(x\right)=\frac{1}{2b}{\left\|u_{b_{i}}-\Phi_{b_{i}}\right\|}_{2}^{2}$. In this case, each sub-function $f_{i}\left(x\right)$ accounts for a collection of observations of size b, rather than only one observation. Thus, when we randomly spilt the smooth function F(x) into multiple sub-functions $f_{i}\left(x\right)$ and block the measurement matrix Φ into multiple sub matrices $\Phi_{b_{i}}$, the computation of the stochastic gradient in the stochastic gradient methods is benefitted.

For the low-rank matrix reconstruction problem, according to the explanation provided in (2) of this section, we know that the sparse basis Ψ consists of infinitely several unit-norm rank-one matrices. According to Equations (5) and (8), the smooth function can be represented as $f_{i}\left(X\right)={\left(u_{i}-\langle \Theta_{i},X\rangle \right)}^{2}$. Therefore, the smooth function F(X) can be written as

(11) $$\begin{array}{ll} F\left(X\right) & =\frac{1}{M}\sum_{i=1}^{M}f_{i}\left(X\right)\;=\;\frac{1}{M}\sum_{i=1}^{M}\left(\frac{1}{2b}\sum_{j=\left(i-1\right)\times b+1}^{ib}{\left(u_{j}-\langle \Phi_{j},X\rangle \right)}^{2}\right)\\ & \overset{\Delta }{=}\;\frac{1}{M}{\sum_{i=1}^{M}\frac{1}{2b}\left\|u_{b_{i}}-\Phi_{b_{i}}*X\right\|}_{2}^{2} \end{array}$$

where M=m/b is the number of block matrix in the sensing matrix Φ and M is an integer. Similarly, each function $f_{i}\left(X\right)$ accounts for a collection of observations $u_{b_{i}}$ of size b, rather than only one observation.

## 3. StoGradMP Algorithm

**Figure 1:**
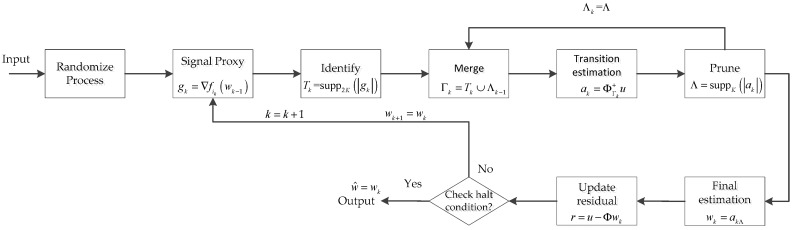
Block diagram of the StoGradMP algorithm.

The CoSaMP [19] algorithm has become popular for reconstructing sparse or compressive signals from their linear non-adaptive measurement. According to the relevant literature, we know that the CoSaMP algorithm is fast for small-scale signals with low dimensionality, but for a large-scale signal with high dimensionality, the reconstruction accuracy and the robustness of the algorithm are considered poor and not ideal. Regarding the shortcomings of the CoSaMP algorithm, Bahmani et al. [28] summarized the idea of the CoSaMP algorithm and proposed a gradient matching pursuit (GradMP) algorithm to solve the reconstruction problem of large-scale class signals with sparsity constraints. However, for large-scale class signals, the GradMP algorithm needs to compute the full gradient of the objective function F(x), which greatly increases the computational cost of the algorithm. Therefore, after the GradMP algorithm, Needell et al. proposed a stochastic version of the GradMP algorithm called StoGradMP [29], which does not need to compute the full gradient of F(x). Instead, at each round of iterations, an index i∈[M] is randomly selected and its associated gradient $f_{i}\left(x\right)$ is computed. This operation is effective for handling the large-scale signal recovery problem, as gradient computation is often prohibitively expensive. To better analyze the StoGradMP algorithm, its block diagram is shown in Figure 1.

The StoGradMP algorithm is described in Algorithm 1, where the steps at each iteration are shown below. Because the reconstruction process of the sparse original signal and the low-rank matrix are almost identical, for the sake of simplicity, we express the above two original signals using w in the subsequent explanations.

**Randomize process**: Randomly determine an index $i_{k}$ with probability $p\left(i_{k}\right)$, where k is the loop index and $i_{k}\in M$. Then, compute its associated block matrix $\Phi_{b_{i}}$ and smooth functions.

**Signal proxy**: Compute the gradient $g_{k}$ of the smooth function, where $g_{k}$ is an n×1 vector. For the low-rank matrix, $g_{k}$ is an n×n matrix.

**Identify**: In compressed sensing, when sorting the absolute values of the gradient in descending order, the first 2K-largest absolute values of the gradient vector are selected. Then, search the atomic index of the block sensing matrix corresponding to these coefficients. Thereafter, a preliminary atomic set $T_{k}$ is formed at the *k*-th iteration. In the low rank matrix reconstruction, the best rank 2R approximation to $g_{k}$ is obtained by keeping the top 2R singular values in the singular value decomposition (SVD).

**Merge:** Establish the candidate atomic set $\Gamma_{k}$ at the *k*-th iteration, which consists of the preliminary atomic set $T_{k}$ at the current iteration and the support atomic set $\Lambda_{k}$ at the previous iteration.

**Estimate:** Calculate the transition signal $a_{k}$ at the current iteration, which is obtained using a sub-optimization method. This is a least squares problem for both the compressed sensing and low-rank matrix reconstruction problems. In compressed sensing, $a_{k}$ is an n×1 vector, whereas in matrix recovery, $a_{k}$ is an n×n matrix.

**Prune:** Sorting the absolute values of the transition signal vector $a_{k}$ in descending order, the first K largest components are selected in vector $a_{k}$, and the atomic index of the candidate atomic set corresponding to these components is then obtained. The support atomic set $\Lambda_{k}$ is constructed at the current iteration. The support atomic set belongs to the candidate atomic set, $\Lambda_{k}\in \Gamma_{k}$. Similarly, in the matrix reconstruction, the best rank R approximation to $a_{k}$ is obtained by retaining the top R singular values in the SVD.

**Update:** Update the current approximate estimation of the original signal, $w_{k}=a_{k\Lambda }$. Here, $\Lambda =\Lambda_{k}$. The position of the nonzero entries of the final estimation signal $w_{k}$ is determined by the index of the support atomic set. $w_{k}$ is the final estimation signal at the *k*th iteration and w represents the original signal, which includes the sparse signal and the low rank matrix signal.

**Check:** When the $l_{2}$-norm of the current residual of the estimation signal $w_{k}$ is smaller than the tolerance error tol_alg or the loop index k is greater than the maximum number of iterations (maxIter), then the reconstruction algorithm halts the iterations and the final approximation estimation of signal $\hat{w}$ is output such that $\hat{w}=w_{k}$. If the halt condition is not satisfied, then the algorithm continues to execute the iterations until the halt condition is met.

The entire procedure is as shown in Algorithm 1.

**Algorithm 1.** StoGradMP Algorithm**Input:**K, u, Φ, p(i)**,**b, tol_alg, maxIter **Output:** an approximation estimation signal $\hat{w}=w_{k}$ **Initialize:**
$\hat{w}=0$, k=0, $\Lambda_{k}=0$, $T_{k}=0$, $\Gamma_{k}=0$, M **repeat** k=k+1 loop index select the $i_{k}$ with probability $p\left(i_{k}\right)$ randomize $g_{k}=\nabla f_{i_{k}}\left(w_{k}\right)$ form signal proxy $T_{k}{=\mathrm{supp}}_{2K}\left(\left|g_{k}\right|\right)$ identify 2K components $\Gamma_{k}=T_{k}\cup \Lambda_{k-1}$ merge to form candidate set $a_{k}=\Phi_{\Gamma_{k}}^{+}u$ transition estimation using least squares method $\Lambda ={\mathrm{supp}}_{K}\left(\left|a_{k}\right|\right)$ prune to obtain the support atomic set $w_{k}=a_{k\Lambda }$ final signal estimation $r=u-\Phi w_{k}$ update the current residual **Until** halting iteration condition is true, exit loop

## 4. Proposed Algorithm

The StoGradMP algorithm takes the sparsity of the original signal as the known information and uses it to complete the expansion of the preliminary atomic set in the preliminary stage of the algorithm. The StoGradMP algorithm determines the 2K-most relevant atoms in the preliminary stage of each round of iterations, and these atoms form a preliminary atomic set. Here, K represents the numerical value of the sparsity level and rank level, which is a fixed number greater than zero. This atomic selection results in the addition of smaller relevant atoms and incorrect atoms to the preliminary atomic set, which reduces the accuracy and speed of the reconstruction algorithm, thereby affecting the reconstruction performance of the algorithm. To solve this problem, we used the weak selection threshold strategy to achieve the expansion of the preliminary atomic set at the preliminary stage of the algorithm.

The entire process explanation of the proposed algorithm is described here. First, according to Equations (10) and (11) in Section 2, we selected the index $i_{k}$ with probability $p\left(i_{k}\right)$. This step is mainly used to randomize the measurement matrix Φ to obtain a stochastic block matrix $\Phi_{b_{i_{k}}}$, which is expressed by

(12) I=ceil(rand×nb)

(13) $$b_{i_{k}}=b\times \left(I-1\right)+1:b\times I$$

where nb is the number of block matrices according to Equation (10), which is equal to nb=floor(m/b). Here, M=nb. b is the number of rows of the block matrix, which is equal to b=min(m,K). When the original signal is a sparse signal, K represents the numerical value of the sparsity level. When the original signal is a low-rank matrix signal, K is the numerical value of the rank level. $b_{i_{k}}$ represents the index of rows of the measurement matrix, which is randomly determined. The block matrix $\Phi_{b_{i_{k}}}$ is also randomly selected. Then, the stochastic gradient function $f_{i}\left(w\right)$ is computed. Here, w consists of the symbols used in Section 3, which represents the sparse original signal and the low rank matrix. According to Equations (10) and (11), the sub-function $f_{i}\left(w\right)$ is expressed as

(14) $$f_{i_{k}}\left(w_{k}\right)=\frac{1}{2b}{\left\|u_{b_{i_{k}}}-\Phi_{b_{i_{k}}}w_{k-1}\right\|}_{2}^{2}$$

where k is the loop index, and $u_{b_{i_{k}}}$ and $\Phi_{b_{i_{k}}}$ are the *i*-th block observation signal and the *i*-th block matrix at k iteration, respectively. From Equations (12)–(14), we know that the sub-function $f_{i}\left(w\right)$ is also stochastically determined, and that $f_{i}\left(w\right)$ belongs to F(w).

When the block matrix $\Phi_{b_{i_{k}}}$ and the stochastic gradient function $f_{i}\left(w\right)$ are obtained, the gradient of sub-function $f_{i}\left(w\right)$ is calculated, which is expressed as

(15) $$g_{k}=\nabla f_{i}\left(w_{k}\right)$$

where $g_{k}$ is the gradient of the sub-function $f_{i}\left(w\right)$ at the k-th iteration, $w_{k}$ is the final estimation of the original signal at the k-th iteration, and ∇(.) denotes the derivative of the sub-function $f_{i}\left(w\right)$. Combining Equation (13) with Equation (14), the gradient $g_{k}$ can be expressed as

(16) $$g_{k}=-2\times \Phi_{b_{i_{k}}}^{T}\left(u_{b_{i_{k}}}-\Phi_{b_{i_{k}}}w_{k-1}\right)$$

where ${\left(.\right)}^{T}$ represents the transpose operation of the matrix.

According to Equation (16), the smaller the absolute value of the gradient, the worse the match between the selected atoms and the original signal. In the StoGradMP algorithm, 2K is fixed and selected as the largest gradient coefficient from the gradient vector $g_{k}$ to determine the atomic index of the block matrix and form the preliminary atomic set. The selected gradient coefficients may contain some smaller gradient coefficients in the StoGradMP algorithm during some iterations. This reduces the reconstruction performance and increases the computational complexity. Therefore, to improve the reconstruction performance of the StoGradMP algorithm, we used the weak selection threshold method to complete the expansion of the preliminary atomic set $T_{k}$. This process can be described as

(17) $$\gamma =\max\left(\left|g_{k}\right|\right)$$

(18) $$T_{k}{=\mathrm{supp}}_{\kappa \times \gamma }\left(\left|g_{k}\right|\right)$$

where γ is the maximum value of the absolute gradient vector $\left|g_{k}\right|$ at the k-th iteration, κ∈[0.1 1.0] is the threshold, and ${\mathrm{supp}}_{\kappa \times \gamma }\left(.\right)$ represents the preliminary atomic set that satisfies the weak selection threshold condition. The gradients corresponding to the preliminary atoms satisfy the condition that their absolute values are greater than κ×γ. If the threshold is greater than 1, then κ×γ is greater than all gradients of the absolute value, and the atomic set is null. This causes the weak selection threshold method to fail. A too-small threshold of κ increases the number of error atoms in the preliminary atomic set. In the low rank matrix reconstruction, the best rank approximation to $g_{k}$ is obtained by maintaining the singular value at a level greater than the weak selection threshold in the SVD, where γ is the maximum singular value. The preliminary atomic set consists of the singular vectors that satisfy the weak selection threshold method.

After selecting the preliminary atomic set, we used it and the previous support atomic set $\Lambda_{k-1}$ to form the current candidate atomic set, which can be expressed as:

(19) $$\Gamma_{k}=T_{k}\cup \Lambda_{k-1}$$

where $\Gamma_{k}$, $T_{k}$, and $\Lambda_{k-1}$ denote the candidate atomic index set, the preliminary atomic index set, and the support atomic index set, respectively.

After the current candidate atomic set was constructed, to ensure the correctness and effectiveness of the proposed method, we added the reliability guarantee method 1 to the proposed algorithm, that is, if

(20) $$\mathrm{supp}\left|\Lambda_{k-1}\right|==\mathrm{supp}\left|\Gamma_{k}\right|$$

is true, where $\mathrm{supp}\left|\Gamma_{k}\right|$ and $\mathrm{supp}\left|\Lambda_{k-1}\right|$ are represents the size (or scale) of the current candidate atomic index set $\Gamma_{k}$ and the previous support atomic index set $\Lambda_{k-1}$, respectively. The method is unable to select the new atoms from the block matrix to add to the candidate atomic set. At this time, the loop is exited and the estimated value of the original signal is the output. We added the sub-condition judgment in the above judgment condition to prevent the proposed method from exiting the loop in the first round of iterations. Since both the candidate atomic set and the support atomic set are empty sets in the first round of iterations, this sub-condition judgment can be expressed as follows: If k==1, then the estimated signal $\hat{w}$ is equal to 0.

Although the weak selection threshold method improves the correlation of the preliminary atomic set and increases the flexibility of atom selection, it is possible that when the threshold is too small, the number of columns of the candidate atomic set is greater than the number of the rows of the candidate atomic set. This leads to an inability to obtain the transition estimation of the original signal using the least squares method because the premise of the least squares method is that the number of rows of the atomic set is greater than the number of columns of the atomic set. Therefore, before solving the least squares method, we must ensure that this condition exists. Therefore, we developed the reliability guarantee method 2, that is, if

(21) $$\mathrm{supp}\left|\Gamma_{k}\right|\leq m$$

then,

(22) $$\Lambda =\Gamma_{k}$$

(23) $$\Phi_{\Lambda }=\Phi \left(:,\Lambda \right)$$

where $\mathrm{supp}\left|\Gamma_{k}\right|$ represents the number of columns of the candidate atomic matrix $\Gamma_{k}$ at the k-th iteration. If this condition is satisfied, then we regard the candidate atomic index set $\Gamma_{k}$ as the current support atomic index set Λ, and the atoms corresponding to the current support atomic index set Λ are used to construct the current support atomic set $\Phi_{\Lambda }$. Conversely, if the condition is not satisfied (the number of rows is smaller than the number of columns), then the matrix ${\left(\Phi_{\Lambda }^{T}\;\times \;\Phi_{\Lambda }\right)}^{-1}$ is not inverse. If this occurs, we exit the loop and let $\hat{w}=0$.

Next, we used the least squares method to solve the sub-optimization problem, which can be described as:

(24) $$a_{k}=\Phi_{{}_{\Lambda }}^{+}u$$

where $a_{k}$ is the transition estimation signal of the original signal, u is the observation signal, and ${\left(\Phi_{\Lambda }\right)}^{+}$ represents the pseudo inverse of the support atomic set $\Phi_{\Lambda }$. To better analyze the role of the reliability guarantee method 2, Equation (24) can be written as:

(25) $$a_{k}={\left(\Phi_{\Lambda }^{T}\times \Phi_{\Lambda }\right)}^{-1}\times \Phi_{\Lambda }^{T}\times u$$

where ${\left(\Phi_{\Lambda }\right)}^{T}$ and ${\left(\Phi_{\Lambda }^{T}\times \Phi_{\Lambda }\right)}^{-1}$ represent the transpose operation and inverse operation of the matrix $\Phi_{\Lambda }$ and the matrix $\Phi_{\Lambda }^{T}\times \Phi_{\Lambda }$, respectively. In combination with Equations (21) and (24), we can ensure that the operation $\Phi_{\Lambda }^{T}\times \Phi_{\Lambda }$ is invertible.

Based on Equations (22)–(24), we observed that the support atomic set is obtained using reliability guarantee method 2 and the candidate atomic set. If the reliability guarantee method 2 is true, then the current candidate atomic set can be regarded as the support atomic set. This operation is used to obtain the final support for the signal estimation. Next, we updated the current residual and final estimation of the original signal, which is expressed as

(26) $$r_{c}=u-\Phi_{\Lambda }a_{k}$$

(27) $$w_{k}=a_{k\Lambda }$$

where $w_{k}$ is the final estimation of the original signal at the k-th iteration, $a_{k\Lambda }$ is the reconstruction signal corresponding to the support atomic index set Λ, and $r_{c}$ is the current residual.

**Figure 2:**
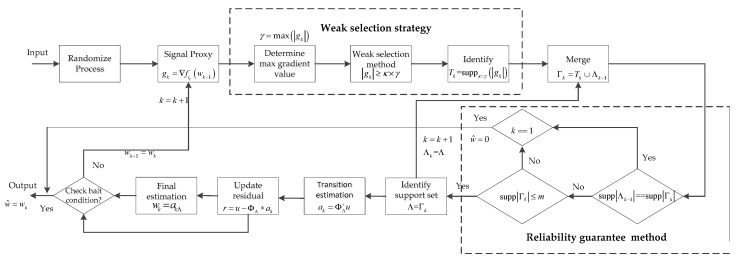
Block diagram of the proposed method.

Finally, for the different original signals, we created different stop iteration conditions if

(28) $${\left\|r_{c}\right\|}_{l}\leq tol\_a\mathrm{lg}\;\mathrm{or}\;k\geq \mathrm{maxIter}$$

is true, where tol_alg is the tolerance error of the algorithm iteration, and maxIter is the maximum number iterations of the algorithm. Specifically, if the original signal is a sparse signal, l=2, that is, the $l_{2}$-norm of the residual estimation vector, then we set tol_alg and maxIter to $1\times 10^{-7}$ and 500×M, respectively. When the original signal is a low-rank matrix, then the current residual estimation is a matrix, which is obtained by conducting a Frobenius norm operation on the error matrix. Here, l=F. We set the tol_alg and maxIter to $1\times 10^{-7}$ and 300×M, respectively. According to Equation (28), when the stop iteration condition is satisfied, the algorithm stops the iterations and the output is the final estimation of the original signal $\hat{w}=w_{k}$. If the halt iteration condition is not satisfied, the iteration is continued, and it updates the current final estimation for the gradient computation of the next iteration, $w_{k+1}=w_{k}$. It continues until the stop iteration condition is true. To better analyze the proposed algorithm, its block diagram is shown in Figure 2.

The entire procedure is shown in Algorithm 2.

**Algorithm 2:** Proposed algorithm.Input: Φ, u, p(i), b, tol_alg, maxIter, κ Output: an approximation estimation signal $\hat{w}=w_{k}$ Initialize: $\hat{w}=0$, k=0, $\Lambda_{k}=0$, $T_{k}=0$, $\Gamma_{k}=0$, M repeat k=k+1 loop index Select $i_{k}$ from [M] with probability $p\left(i_{k}\right)$ randomize process $g_{k}=\nabla f_{i_{k}}\left(w_{k-1}\right)$ form signal proxy $\gamma =\max\left(\left|g_{k}\right|\right)$ determine the max gradient value $T_{k}={\mathrm{supp}}_{\kappa \times \gamma }\left(\left|g_{k}\right|\right)$ weak selection method to identify the preliminary atomic set $\Gamma_{k}=T_{k}\cup \Lambda_{k-1}$ merge to form candidate set **Reliability guarantee method 1** If $\mathrm{supp}\left|\Lambda_{k-1}\right|==\mathrm{supp}\left|\Gamma_{k}\right|$  If k==1    $\hat{w}=0$;  end  break; end **Reliability guarantee method 2** If $\mathrm{supp}\left|\Gamma_{k}\right|\leq m$  $\Lambda =\Gamma_{k}$ identify the support atomic set  $\Phi_{\Lambda }=\Phi \left(:,\Lambda \right)$ else  If k==1    $\hat{w}=0$;  end  break; end $a_{k}=\Phi_{\Lambda }^{+}u$ transition estimation by least squares method $r=u-\Phi_{\Lambda }a_{k}$ update the current residual $w_{k}=a_{k\Lambda }$ final signal estimation Until halting iteration condition is true, exit loop

## 5. Discussion

We analyzed the simulation for the following experiments: 1D sparse signal reconstruction, low rank matrix reconstruction, and 2D image signal reconstruction. The reconstruction performance is an average after running the simulation 200 times using a computer with a quad-core, 64-bit processor, and 4G memory.

### 5.1. 1D Sparse Signal Reconstruction Experiment

In this experiment, we used a random signal with K-sparse as the original signal. The measurement matrix was randomly generated with a Gaussian distribution. We set the range of the weak selection thresholds κ to [0.2,0.4,0.6,0.8]. The recovery error and iteration stop error of all the algorithms were set to $1\times 10^{-6}$ and $1\times 10^{-7}$, respectively. These errors were obtained by conducting an $l_{2}$-norm operation on the error vector. The maximum number of iterations maxIter was set to 500×M.

**Figure 3:**
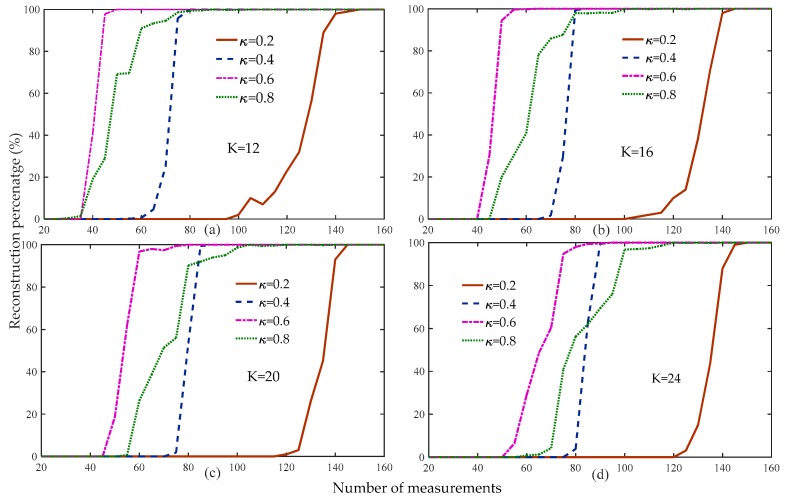
Reconstruction percentage of the proposed method at different weak selection thresholds (n=256, K=12,16,20,24, κ=0.2,0.4,0.6,0.8, m=20:5:160, Gaussian signal).

Figure 3 compares the reconstruction percentage of the proposed algorithm to the different thresholds. Figure 3 shows that when the threshold was 0.6, the reconstruction percentage of the proposed algorithm was the highest compared to the other thresholds under the same measurements and sparsity levels.

**Figure 4:**
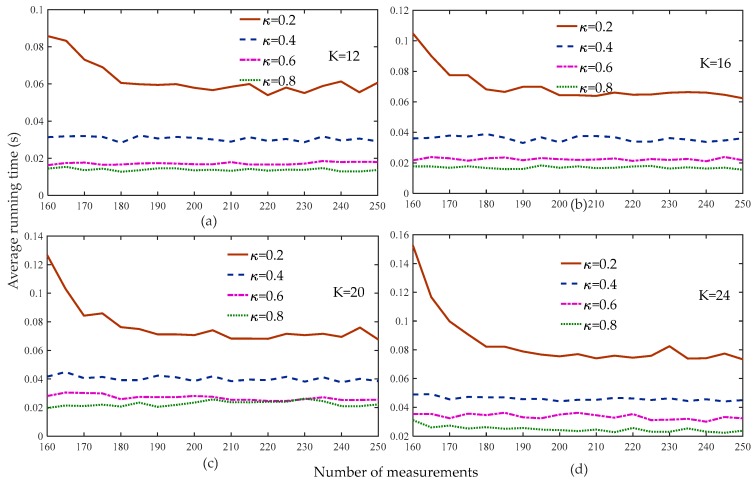
Average running time of the proposed method with different weak selection thresholds (n=256, K=12,16,20,24, κ=0.2,0.4,0.6,0.8, m=160:5:250, Gaussian signal).

Figure 3 shows that the reconstruction percentage of the proposed algorithm was 100% for all of the sparsity and threshold levels when the number of measurements was greater than 160. Therefore, we set the range of measurements to [160 – 250] to compare the average running time of the proposed algorithm at different weak selection thresholds, as shown in Figure 4. Figure 4 shows that the average running time of the proposed algorithm was the shortest for different sparse levels when the threshold was 0.8, followed by 0.6, with very small differences between the two. Based on the analysis of Figure 3 and Figure 4, we set the default weak selection threshold to 0.6.

**Figure 5:**
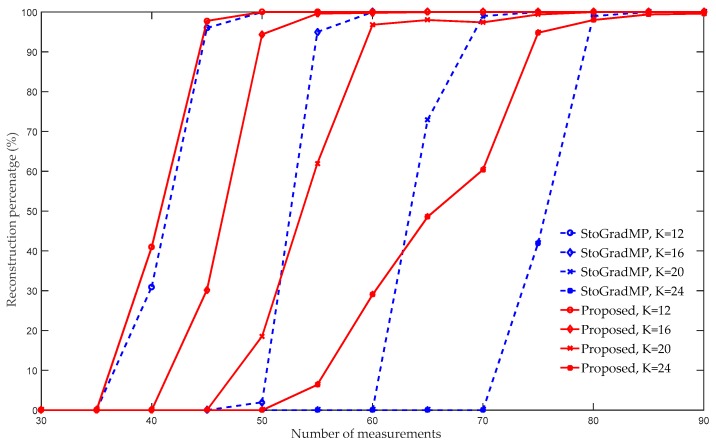
Reconstruction percentages of the StoGradMP and proposed algorithms (n=256, κ=0.6, K=12,16,20,24, m=20:5:90, Gaussian signal).

Figure 5 compares the reconstruction percentage of the proposed algorithm using the StoGradMP algorithm. We set the sparse level to K∈[12,16,20,24], and the weak selection threshold to 0.6. Figure 5 shows that when the sparse level was 12, the reconstruction percentages of the proposed algorithm and the StoGradMP algorithm were nearly identical for all the measurements. When K=16, 20, or 24, the reconstruction percentage of the proposed algorithm was higher than that of the StoGradMP algorithm. When the sparse level was 24, the difference in the reconstruction percentages between the two algorithms was the largest. Therefore, we concluded that when the sparse level increases, the difference between the reconstruction percentages increases further. This means that in sparse signal reconstruction, the proposed method is more suitable for reconstruction in a larger sparsity environment compared to the StoGradMP algorithm.

**Figure 6:**
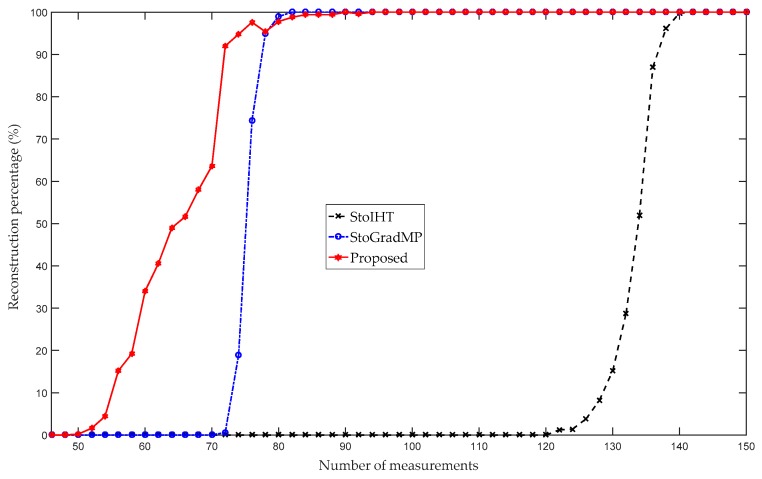
Reconstruction percentages of the different algorithms (n=256, K=24, κ=0.6, m=46:2:150, Gaussian signal).

Figure 6 compares the reconstruction percentage of the proposed algorithm with the StoGradMP and StoIHT algorithms. In Figure 5, the interval of measurement was set to five, and to reflect the details, we set the interval of measurement to two in Figure 4. Figure 6 shows that when 46≤m<50, the reconstruction percentages of the methods were 0%. This means that they could not complete the reconstruction. When 50≤m≤76, the reconstruction percentage of the proposed method ranged from 0.2% to 97.6%; however, the reconstruction percentages of the StoGradMP and StoIHT algorithms were still 0%. When 72≤m≤78, the reconstruction percentage of the StoGradMP algorithm began to increase from 0.6% to 95%. When 78≤m≤120, the reconstruction percentages of the proposed and StoGradMP algorithms were nearly 100%. However, the StoIHT algorithm was still unable to complete reconstruction. When 120≤m≤142, the reconstruction percentage of the StoIHT algorithm increased from 0% to 100%. When 142≤m, then all the reconstruction algorithms could achieve full reconstruction. This demonstrates that the proposed method provides better reconstruction performance than the others.

**Figure 7:**
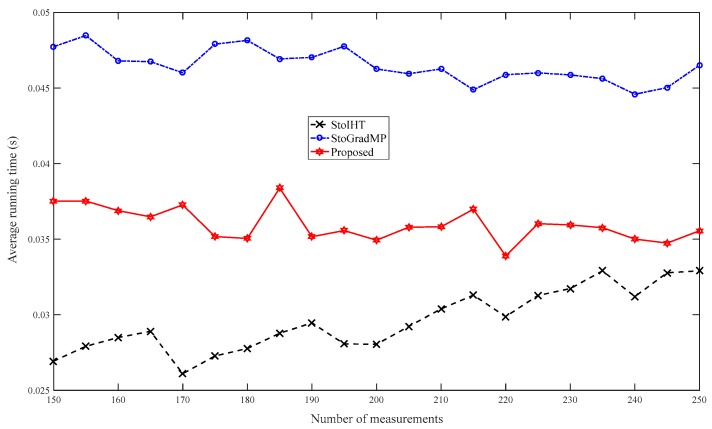
Average running times of the different algorithms (n=256, K=24, κ=0.6, m=150:5:250, Gaussian signal).

Figure 7 compares the average running time. Figure 6 shows that the reconstruction percentage was 100% for all the reconstruction algorithms when the number of measurements was greater than 150. Therefore, in this simulation, we set the range of measurement to [150– 250]. Figure 7 shows that the proposed method has a shorter running time than the StoGradMP algorithm. Although the StoIHT algorithm had a shorter running time than the other algorithms, it required more measurements to achieve the same reconstruction percentage as the other algorithms.

**Figure 8:**
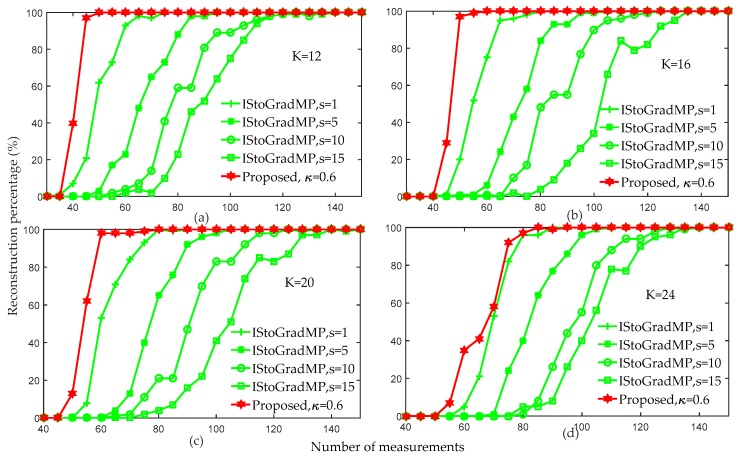
Reconstruction percentages of the proposed algorithm and IStoGradMP algorithm (n=256, κ=0.6, K=12,16,20,24, s=1,5,10,15, m=30:5:150, Gaussian signal).

Figure 8 compares the reconstruction percentages of the proposed algorithm and the prior improved algorithm (IStoGradMP) [33]. Both of these algorithms reconstructed the signal in an unknown sparsity environment. The main differences between the proposed algorithm and the IStoGradMP algorithm are: (1) In the preliminary atomic stage, the proposed algorithm uses the atomic matching strategy to obtain the preliminary atomic set, whereas the IStoGradMP algorithm evaluates and adjusts the estimated sparsity of the original signal to obtain the preliminary atomic set; (2) the scales of the preliminary atomic set and the support atomic set are unfixed at each iteration in the proposed algorithm, whereas the scale of the preliminary atomic set and the support atomic set are fixed at each iteration of the IStoGradMP algorithm; and (3) the support atomic set is determined by the candidate atomic set and the reliability guarantee method for the proposed method, whereas the support atomic set is determined by pruning the candidate atomic set in the IStoGradMP algorithm. We also proposed an improved StoGradMP algorithm based on the soft-threshold method [34]. This algorithm [34] requires that sparsity information of the original signal to be known, whereas the proposed method and the IStoGradMP algorithm [33] can reconstruct the signal without knowing the sparsity information. Based on the above comparative analysis, in this section, we only compared the experimental simulations in an unknown sparsity environment. We only compared the IStoGradMP and the proposed methods.

Figure 8 shows that for arbitrary measurement values, the reconstruction percentage of the proposed algorithm was higher than that of the IStoGradMP algorithm. However, we discovered that as the sparsity level increased, the gap in the reconstruction percentage between the proposed algorithm and the IStoGradMP algorithm gradually reduced. This means that the proposed method is more suitable than the IStoGradMP algorithm under smaller sparsity environments.

**Figure 9:**
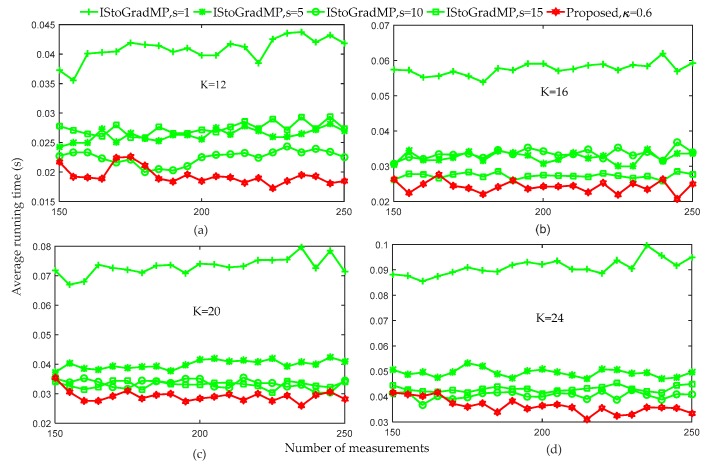
Average running times of the proposed algorithm and IStoGradMP algorithm (n=256, κ=0.6, K=12,16,20,24, s=1,5,10,15, m=150:5:250, $\delta_{K}=0.1$, Gaussian signal).

Figure 9 compares the average running times of the proposed method and the IStoGradMP method under different sparsity level conditions. Figure 8 shows that when the number of the measurements was greater than 150, the reconstruction percentage of the proposed method and the IStoGradMP was 100% for all the sparsity levels. Therefore, we set the range of the number of the measurement to [150 – 250]. Figure 9 shows that when the threshold of the proposed method was set to 0.6, the average running time of the proposed method was less than that for the IStoGradMP algorithm. This means that the computational complexity of the proposed algorithm was lower than that for the IStoGradMP algorithm. That is, the proposed method was faster than the IStoGradMP algorithm under the full reconstruction conditions.

Based on the analysis of Figure 8 and Figure 9, we conclude that for a smaller sparsity level environment, the proposed algorithm with a proper weak selection threshold has better reconstruction performance as well as a lower computational complexity than the IStoGradMP algorithm.

### 5.2. Low-Rank Matrix Reconstruction Experiment

In this experiment, we used the random matrix with a low-rank property as the original signal. We set the rank level of the matrix to R=1,3. The size of the low rank matrix was 10×10. The measurement matrix was randomly generated with a Gaussian distribution. The recovery error and iteration halt error of all the algorithms were set to $1\times 10^{-6}$ and $1\times 10^{-7}$, respectively. These errors were obtained using a Frobenius norm operation on the respective error matrix. The maximum number of iterations maxIter was set to 300×M.

**Figure 10:**
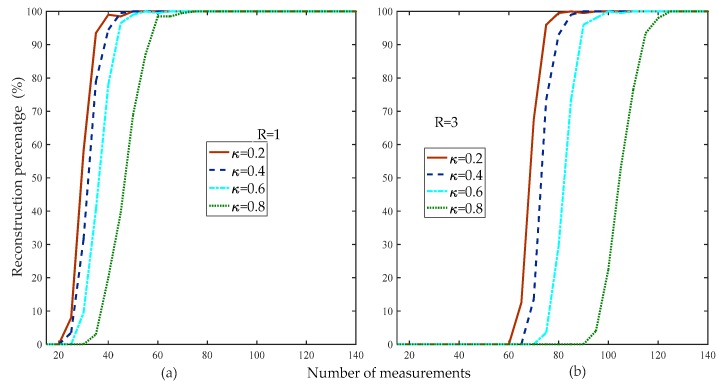
Reconstruction percentage of the proposed method with different weak selection thresholds (d=100, R=1,3, κ=0.2,0.4,0.6,0.8, m=15:5:140, random low rank matrix).

Figure 10 compares the reconstruction percentage of the proposed method at different weak selection thresholds. Figure 10 shows that the reconstruction percentage was higher than the other algorithms when the threshold was 0.2 for the different rank levels of the matrix.

**Figure 11:**
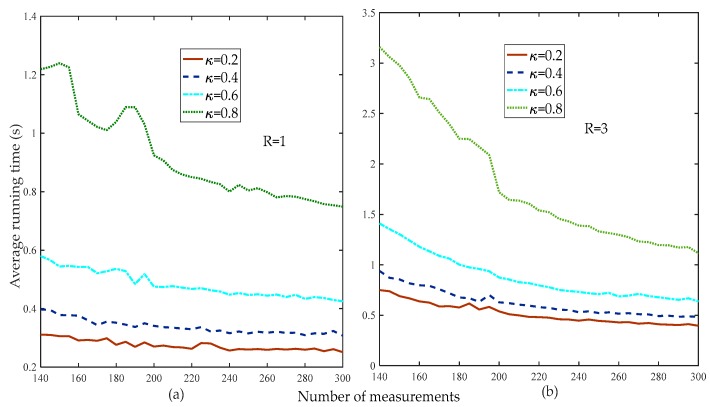
Average running times of the proposed method at different weak selection thresholds (d=100, R=1,3, κ=0.2,0.4,0.6,0.8, m=200:5:300, random low-rank matrix).

Figure 11 compares the average running times of the proposed method at different thresholds. Figure 11 shows that the smaller the weak selection threshold, the higher the reconstruction percentage, which means that a larger threshold increases the computational complexity of the proposed method. Thus, in the subsequent simulation without special instructions, the default weak selection threshold was set to 0.2.

**Figure 12:**
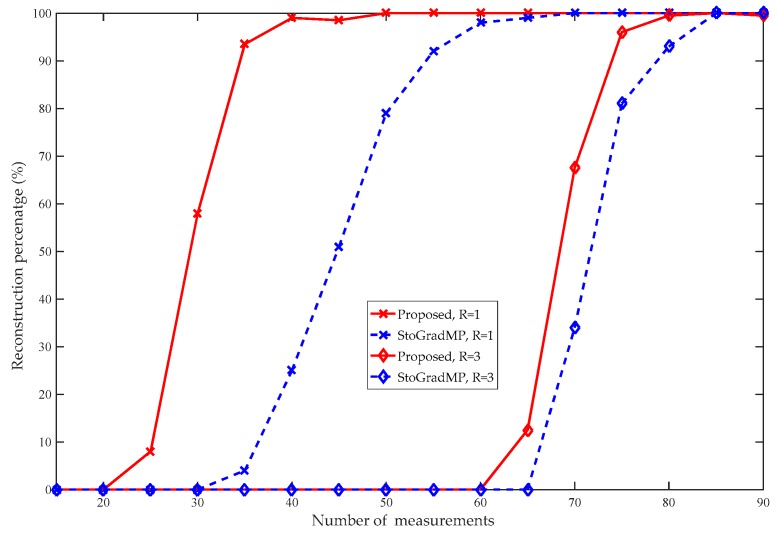
Reconstruction percentages of the StoGradMP and proposed algorithms (d=100, R=1,3, κ=0.2, m=15:5:120, random low-rank matrix).

Figure 12 compares the reconstruction percentages of the different measurements of the proposed and StoGradMP algorithms at different rank levels. We observed that the proposed method had a better reconstruction percentage than the StoGradMP algorithm at the different rank levels.

**Figure 13:**
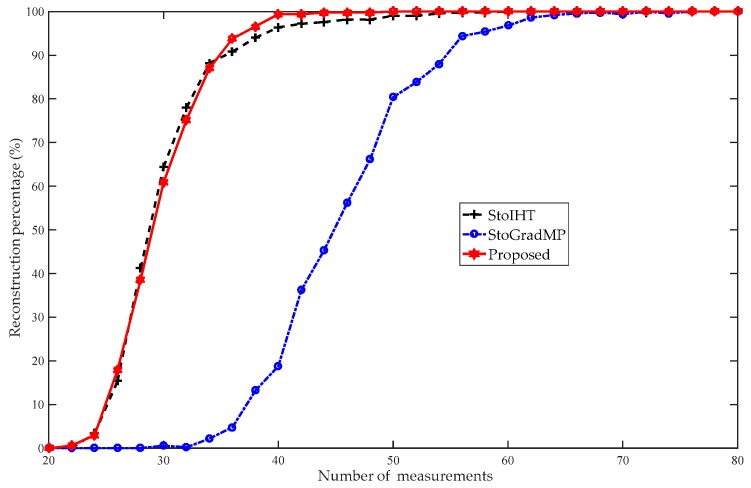
Reconstruction percentages of the different methods (d=100, R=1, κ=0.2, m=20:2:70, randomly low-rank matrix).

Figure 13 compares the reconstruction percentage of the proposed algorithm to the StoGradMP and StoIHT algorithms for different measurements. In Figure 12, we set the measurement interval to five, and to show details, we set the interval of measurement to two in Figure 13. Figure 13 shows that when 20≤m<22, the reconstruction percentage of all the algorithms was 0%. When 22≤m≤34, the reconstruction percentage of the StoIHT and proposed algorithms began to increase from 0% to 88.2% and 0.6% to 87%, respectively. However, the StoGradMP algorithm struggled to complete the signal reconstruction. When 32≤m≤58, the reconstruction percentage of the proposed algorithm ranged approximately from 87% to 100%. The reconstruction percentage of the StoIHT algorithm increased from 88.2% to 99.8%. The reconstruction percentage of the StoGradMP algorithm increased from 0.2% to 95.4%. In this measurement range, the reconstruction percentage of the proposed algorithm was higher than those of the other algorithms. When 58≤m, almost all the reconstruction algorithms achieved a high probability reconstruction. Therefore, we conclude that the reconstruction percentages of the proposed method and the StoIHT method were almost the same, and higher than the StoGradMP algorithm, which existed at a lower rank level of the matrix.

**Figure 14:**
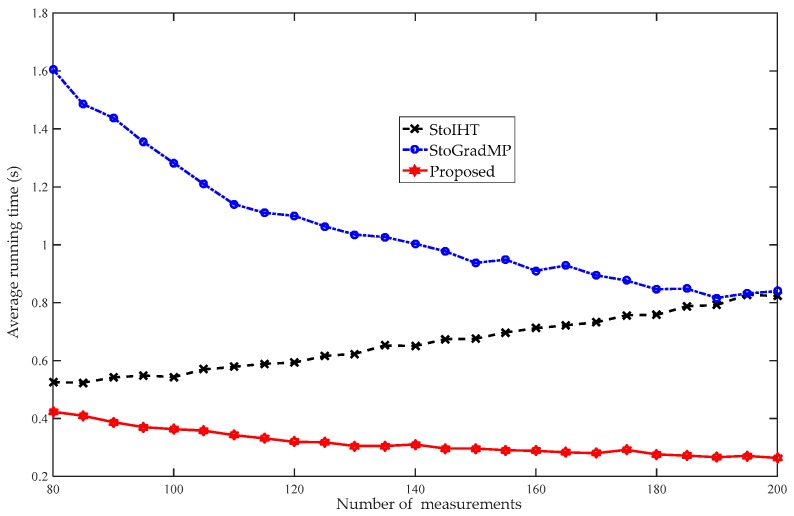
Average running times of the different algorithms (d=100, R=1, κ=0.2, m=80:5:200, randomly low-rank matrix).

Figure 14 compares the average running times of the different algorithms. Figure 13 shows that when the number of measurements was more than 80, the reconstruction percentage of all the algorithms was 100%. Therefore, to better analyze the computational complexity of the different algorithms, we set the range of measurements to [80 – 200] in the simulation. Figure 14 shows that the proposed algorithm had the shortest running time, followed by the StoIHT and StoGradMP algorithms. We observed that when the number of measurements increased, the running time of the StoIHT algorithm also increased, whereas the average running times of the proposed and StoGradMP algorithms tended to decrease and remain stable.

Based on the above analysis, we conclude that the proposed algorithm with a weak selection threshold produces better reconstruction performance than the StoGradMP algorithm, as well as a lower computational complexity than the other algorithms.

### 5.3. 2D Image Signal Reconstruction Experiment

In this subsection, we used six 256×256 test images as the original images. We considered the image signal as a 2D signal. The test images included the following: Baboon, Boat, Cameraman, Fruits, Lena (human portrait), and Peppers. The sparse basis was a wavelet basis with sparse representation capability, and the size was 256×256. The measurement matrix was randomly generated with a Gaussian distribution, and the size was 153×256. We assumed that the sparsity was 51. The iteration halt error of the algorithm was set to $1\times 10^{-7}$, the maximum number of iterations maxIter was set to 30, and the weak selection threshold was set to κ=0.6.

We used the peak signal to noise ratio (PSNR) as an indicator to evaluate the reconstruction quality, which could be expressed as:

(29) $$MSE=\frac{1}{M\times N}\sum_{i=0}^{M-1}\sum_{j=0}^{N-1}{\left|\hat{x}\left(i,j\right)-x\left(i,j\right)\right|}^{2}$$

(30) $$PSNR=10\times \log_{10}\left(\frac{MAX_{\hat{x}}^{2}}{MSE}\right)=20\times \log_{10}\left(\frac{MAX_{\hat{x}}}{\sqrt{MSE}}\right)$$

where M=N=256; $\hat{x}\left(i,j\right)$ and x(i,j) represent the reconstruction value and the original value of the correspondence position, respectively; MSE is the mean square error; and $MAX_{\hat{x}}$ represents the maximum value of the color of the image point. In this paper, each sample point is represented by eight bits, $MAX_{\hat{x}}=255$. The larger the PSNR, the higher the reconstructed image quality.

**Figure 15:**
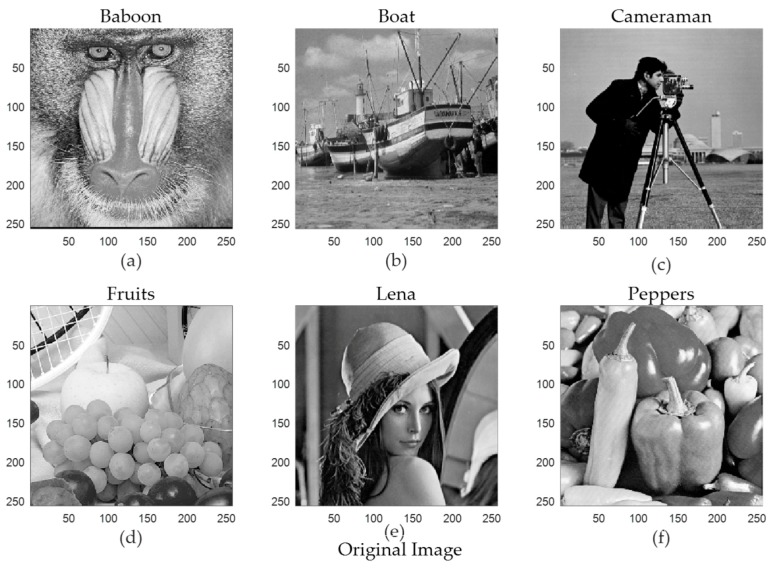
Original images (**a**) Baboon image, (**b**) Boat image, (**c**) Cameraman image, (**d**) Fruits image, (**e**) Lena image, (**f**) Peppers image.

**Figure 16:**
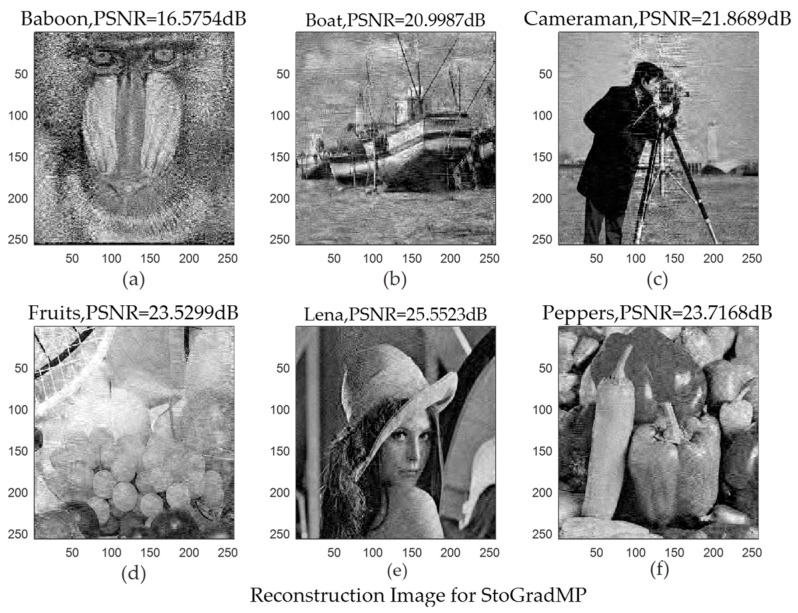
Reconstructed images using the StoGradMP algorithm (**a**) Reconstructed Baboon image with PSNR = 16.5754 dB; (**b**) Reconstructed Boat image with PSNR = 20.9987 dB; (**c**) Reconstructed Cameraman image with PSNR = 21.8698 dB; (**d**) Reconstructed Fruits image with PSNR = 23.5299 dB; (**e**) Reconstructed Lena image with PSNR = 25.5532 dB; (**f**) Reconstructed Peppers image with PSNR = 23.7168 dB.

**Figure 17:**
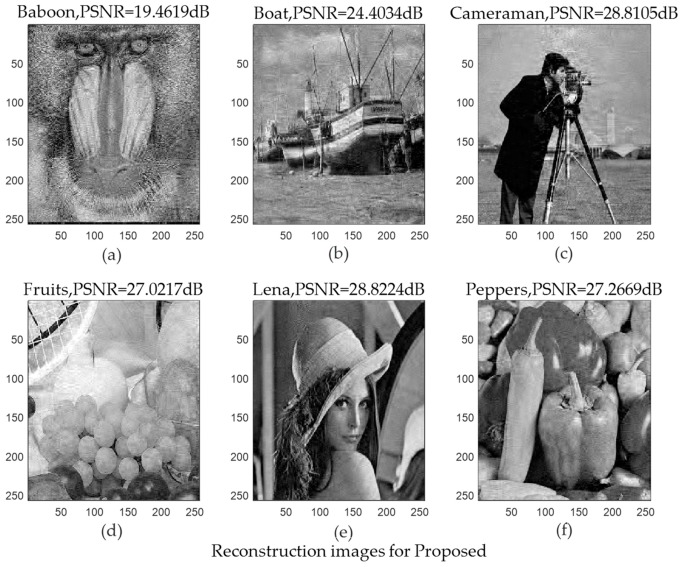
Reconstructed images using the proposed algorithm (**a**) Reconstructed Baboon image with PSNR = 19.4619 dB; (**b**) Reconstructed Boat image with PSNR = 24.4034 dB; (**c**) Reconstructed Cameraman image with PSNR = 28.8105 dB; (**d**) Reconstructed Fruits image with PSNR = 27.0217 dB; (**e**) Reconstructed Lena image with PSNR = 28.8224 dB; (**f**) Reconstructed Peppers image with PSNR = 27.2669 dB.

Figure 15 shows the original images. Figure 16 and Figure 17 shows the reconstructed images using the StoGradMP and proposed algorithms, respectively. Comparing the reconstructed images to the original images, we observed that the two methods successfully reconstructed the original images.

Table 1 compares the average PSNR of the StoGradMP and proposed algorithms under different test image conditions. Table 1 shows that the average PSNR of the proposed algorithm was higher than the StoGradMP algorithm for the different test images, and the average PSNR of the proposed method was higher than 3–4 dB. This shows that the reconstructed image quality of the proposed algorithm was better than the StoGradMP.

Table 2 compares the average running times of the StoGradMP and proposed algorithms for the different test images. From Table 2, the average running times of the StoGradMP algorithm was longer than the proposed method for the different test images, and the average running time of the StoGradMP algorithm was more than twice that of the proposed algorithm. This means that the proposed method had lower computational complexity than the StoGradMP algorithm when images were reconstructed.

Based on the above analysis, the proposed method has a better reconstruction performance for different test images compared to the StoGradMP algorithm, as well as a lower computational complexity than the StoGradMP algorithm.

## 6. Conclusions

In this paper, a novel stochastic gradient matching pursuit algorithm based on weak selection thresholds was proposed. This algorithm uses the weak selection threshold method to select the atoms that best match the original signal from the block sensing matrix and completes the expansion of the preliminary atomic set. The proposed algorithm adopts two reliability guarantee methods to identify the support atomic set and calculate the transition estimation of the original signal to ensure the correctness and effectiveness of the proposed algorithm. The proposed algorithm not only eliminates dependency on prior sparsity information of the original signal, but also increases the flexibility of the atomic selection process while improving atomic reliability. Therefore, it enhances the reconstruction accuracy and reconstruction efficiency of the proposed algorithm.

Our series of simulation results showed that the proposed method has better reconstruction performance and less computational complexity compared to the other algorithms. Future research should consider using the proposed method to process large-scale array signals, such as wireless communication signals, radar signals, and sonar signals, to enhance the useful signal, suppress noise interference, reduce the burden on sensor devices, and ensure fast real-time transmission of the array signal. The weak selection threshold was determined by setting the threshold and the maximum stochastic gradient in our proposed method. The optimal setting threshold was different for different types of signals, which affects the reconstruction performance. Our future work will consider methods to adapt the setting threshold to the signal.

## Figures and Tables

**Table 1:** Comparison of the average peak signal to noise ratios (PSNR) of the StoGradMP and proposed algorithms for the different test images.

	Algorithm	
Image	StoGradMP	Proposed
Baboon	16.5263 dB	19.4775 dB
Boat	20.9818 dB	24.2238 dB
Cameraman	22.3862 dB	25.7790 dB
Fruits	23.3538 dB	26.9003 dB
Lena	25.2590 dB	28.7027 dB
Peppers	23.7567 dB	27.2286 dB

**Table 2:** Comparison of the average runtimes of the StoGradMP and proposed algorithms for the different test images.

	Algorithm	
Image	StoGradMP	Proposed
Baboon	51.04 s	19.32 s
Boat	51.45 s	20.22 s
Cameraman	51.13 s	17.94 s
Fruits	81.66 s	19.41 s
Lena	50.52 s	22.64 s
Peppers	56.88 s	19.03 s
